# Solution Structure of the *Cutibacterium acnes*-Specific Protein RoxP and Insights Into Its Antioxidant Activity

**DOI:** 10.3389/fcimb.2022.803004

**Published:** 2022-02-11

**Authors:** Kristian Stødkilde, Jakob Toudahl Nielsen, Steen Vang Petersen, Bernhard Paetzold, Holger Brüggemann, Frans A. A. Mulder, Christian Brix Folsted Andersen

**Affiliations:** ^1^ Department of Biomedicine, Aarhus University, Aarhus, Denmark; ^2^ Interdisciplinary Nanoscience Center (iNANO), Aarhus University, Aarhus, Denmark; ^3^ S-Biomedic, Beerse, Belgium

**Keywords:** *Cutibacterium acnes*, RoxP, nuclear magnetic resonance, immunoglobin-like, antioxidant

## Abstract

*Cutibacterium acnes* is a predominant bacterium on human skin and is generally regarded as commensal. Recently, the abundantly secreted protein produced by *C. acnes*, RoxP, was shown to alleviate radical-induced cell damage, presumably *via* antioxidant activity, which could potentially be harnessed to fortify skin barrier function. The aim of this study was to determine the structure of RoxP and elucidate the mechanisms behind its antioxidative effect. Here, we present the solution structure of RoxP revealing a compact immunoglobulin-like domain containing a long flexible loop which, in concert with the core domain, forms a positively charged groove that could function as a binding site for cofactors or substrates. Although RoxP shares structural features with cell-adhesion proteins, we show that it does not appear to be responsible for adhesion of *C. acnes* bacteria to human keratinocytes. We identify two tyrosine-containing stretches located in the flexible loop of RoxP, which appear to be responsible for the antioxidant activity of RoxP.

## Introduction

The skin is the human body’s largest organ and inhabited by millions of bacteria (Grice and Segre, 2011; Byrd et al., 2018). These bacteria have been extensively studied in the context of disease; however, only recently have their impact on maintaining a healthy skin been acknowledged (Sanford and Gallo, 2013; Schommer and Gallo, 2013; Belkaid and Hand, 2014; Christensen and Bruggemann, 2014; Belkaid and Tamoutounour, 2016; Chen et al., 2018). Indeed, some skin bacteria formerly described as commensals are now thought to exist in a mutualistic relationship with the host (Wang et al., 2014; Keshari et al., 2020; O'Neill et al., 2020). One such bacterium is *Cutibacterium acnes* (formerly known as *Propionibacterium acnes*). *C. acnes* is an anaerobic, gram-positive bacterium that resides deep in the sebaceous follicles. The normal skin is colonized by a multitude of different strains of *C. acnes* that belong to different phylotypes, designated IA_1_, IA_2_, IB, IC, II, III (McLaughlin et al., 2019). Phylotype diversity is associated with healthy skin, while *acne vulgaris*-affected skin is characterized by loss of diversity and the relative predominance of a few phylotypes, in particular strains of phylotypes IA_1_ and IA_2_ (Nakase et al., 2017; Dagnelie et al., 2018; McLaughlin et al., 2019). Recent studies suggest, that *C. acnes* may play a role in maintaining redox homeostasis *via* secretion of a protein coined RoxP (Radical oxygenase of *Propionibacterium acnes*) (Allhorn et al., 2016; Andersson et al., 2019). RoxP is a stable, 16 kDa protein reported to bind heme and readily reduce 2,2’-azino-bis(3-ethylbenzothiazoline-6-sulfonic acid) radicals (ABTS^+*^) (Allhorn et al., 2016). Whether RoxP features an actual heme-binding site or the interaction is caused by the inherent stickiness of heme is unsettled. Additionally, it is not known if the antioxidant activity is connected to the putative heme-binding, is facilitated through a yet-to-be-found cofactor or by RoxP itself. All Cutibacterium species express catalase and several also superoxide dismutase (Tally et al., 1977; Yorozu et al., 2015; Yamamoto et al., 2019), which are both intracellular antioxidants. RoxP on the other hand harbors an N-terminal signal peptide for secretion (Holland et al., 2010; Allhorn et al., 2016). RoxP is the protein most abundantly secreted by *C. acnes* (Holland et al., 2010; Yu et al., 2016; Jeon et al., 2017), indicative of a functional importance for the bacterium. Although primarily found outside the bacterial cell, RoxP has also been reported to be the second-most abundant cell surface-associated protein (Yu et al., 2015). RoxP can protect monocytic THP-1 cells and keratinocytic HaCaT cells from damage when they are exposed to the reactive oxygen species (ROS)-generating compound paraquat (Andersson et al., 2019). This suggests that RoxP may protect the bacteria, and concomitantly the human host, from free radicals induced by UV-light, pollution or aging cells. The amino acid sequence of RoxP is almost fully conserved between all known *C. acnes* phylotypes and the closely related *Cutibacterium humerusii* (Allhorn et al., 2016), but not found in other cutaneous *Cutibacterium* species, including *Cutibacterium avidum* and *Cutibacterium granulosum* (Mak et al., 2013). Sequence analysis of RoxP identifies no homologs outside the *Cutibacterium* genus and thus bioinformatic approaches do not provide hints to its structure or function. To shed light on the function of RoxP, we determined its solution structure by NMR spectroscopy and combined this with data on its antioxidant activity. Integrating these results, we discuss the function of RoxP, its putative role as an antioxidant, and possible beneficial effects to human skin.

## Materials and Methods

### Protein Expression and Purification

The gene encoding RoxP (residues 24-161) was amplified (primers: 5’-CCGGCGATGGC CATGACACCCATCGATGAGAGCCAACTT-3’ and 5’- GTGGTGGTGCTCGAGGTTGA GGGCGGGATCCACCATGAAT-3’) and cloned into the NcoI and XhoI sites of the Novagen pET22b(+) vector (Merck, Darmstadt, Germany, Catalog-number: 69744), thereby adding a leucine and glutamic acid residue C-terminally before the vector-encoded tag of six histidines. For expression of non-labelled RoxP, Novagen competent BL21 (DE3) cells (Merck, Darmstadt, Germany, Catalog-number: 69450) were transformed and a single colony was grown overnight at 37°C in 50 mL LB medium with 50 μg/mL ampicillin. The pre-culture was then diluted 1:100 into 500 mL LB medium with 50 μg/mL ampicillin. At an OD600 of 0.6, the temperature was lowered to 15°C and protein expression was induced with 0.1 mM isopropyl beta-D-1-thiogalactopyranoside (IPTG) (Merck, Darmstadt, Germany, Catalog-number:11411446001). After overnight incubation, the cells were harvested by centrifugation at 6,000 x *g* for 30 min and stored at -80°C.

For expression of ^13^C/^15^N-labelled RoxP, the pre-culture was diluted 1:200 into 500 mL isotope-growth medium (1 mM MgSO_4_, 0.3 mM CaCl_2_, 1 μg/mL biotin, 1 μg/mL thiamin, 2 mg/mL ^13^C-glucose (Merck, Darmstad, Germany, Catalog-number: 389374), 0.05 mg/mL EDTA, 8.3 μg/mL FeCl_3_, 0.84 μg/mL ZnCl_2_, 0.13 μg/mL CuCl_2_, 0.10 μg/mL CoCl_2_, 0.10 μg/mL H_3_BO_3_, 0.16 μg/mL MnCl_2_, 6 mg/mL Na_2_HPO_4_, 3 mg/mL KH_2_PO_4_, 0.5 mg/mL NaCl, 0.5 mg/mL ^15^NH_4_Cl (Merck, Darmstad, Germany, Catalog-number: 609471) and 50 μg/mL ampicillin, pH 7.2). Protein expression was induced at an OD600 of 0.5 with 0.1 mM IPTG. After overnight expression, cells were harvested by centrifugation for 30 min at 4,000 x *g* and stored at -80°CC. Cell pellets were resuspended in 20 mL ice-cold TES buffer (200 mM Tris-HCl, 0.5 mM ethylenediaminetetraacetic acid (EDTA), 0.5 M sucrose pH 8.0) and incubated on ice for 60 minutes on an orbital shaker. 40 mL ice-cold TES buffer diluted 1:4 in H_2_O was added and incubation was continued for an additional 45 minutes. The osmotically-shocked cells were then centrifuged for 20 minutes at 10,000 x *g* at 4°C and the supernatant, containing the periplasmic content, recovered. The periplasmic extract was subsequently loaded onto a 5 mL HisTrap FF crude column (Cytiva, MA 01752, USA, catalog-number: 17528601) equilibrated in 500 mM NaCl, 20 mM imidazole, 20 mM Tris-HCl pH 7.6. Step-elution was performed in an identical buffer but with 500 mM imidazole. A final purification step of size-exclusion chromatography was applied using a Superdex75 column (Cytiva, MA 01752, USA, catalog-number: 17517401) equilibrated with either 137 mM NaCl, 2.7 mM KCl, 8.1 mM Na_2_HPO_4_, 1.8 mM KH_2_PO_4_, pH 7.4 for non-labelled protein or 50 mM KCl, 20 mM NaH_2_PO_4_ pH 5.8 for ^13^C/^15^N-labelled protein.

### Structure Determination

NMR data were acquired at 298 K on a Bruker 950 spectrometer with a cryogenic probe head. The NMR sample has a 1.2 mM sample concentration. The following experiments were recorded: 2D ^1^H-^13^C HSQC, 2D ^1^H-^15^N HSQC, 3D HNCO, 2D ^1^H-^15^N TROSY-HSQC, 2D HBCBCGCDHD, 2D HBCBCGCDCEHE, 3D HNCACB, 3D H(CCO)HN, 3D HN(CA)CO, 3D C(CO)NH, 3D (H)CCH-TOCSY, 3D ^15^N-separated NOESY, 3D ^1^H-^13^C NOESY aliphatic, and 4D ^13^C-separated NOESY.

The resonances were assigned using standard procedures and the FLYA software (Schmidt and Guntert, 2012) based on a series of different 3D multinuclear NMR experiments. The structure was calculated using distance constraints derived from 3D ^13^C-HSQC-NOESY and ^15^N-HSQC-NOESY spectra and dihedral angle constraints derived from the assigned chemical shift using TALOS-N (Shen and Bax, 2015) The torsion angle predictions classified as “good” by TALOS-N were applied yielding 182 torsion angle restraints. The automatic simultaneous iterative structure refinement and NOE assignment within Cyana (Guntert, 2004) was applied to calculate an ensemble of 20 lowest-energy structures derived from 3159 distance constraints from which 1397 were long-range (9.8 constraints per residue). The quality of the structure is high as evaluated using Cyana and the Protein Structure Validation Suite (psvs-1_5-dev.nesg.org) (Bhattacharya et al., 2007). All protein figures were prepared in PyMOL (Schrodinger, 2015)

### UV-VIS Analysis

RoxP (62.5 μM) in PBS was mixed with either 10 μM hemin (Merck, Darmstad, Germany, Catalog-number: H9039) or 10 μM protoporphyrin IX (Merck, Darmstad, Germany, Catalog-number: P8293) and incubated for one hour at 25°CC. Subsequently UV-VIS spectra from 190 nm to 840 nm was recorded on a Nanodrop 2000c (ThermoFisher Scientific, MA 02451, USA, Catalog-number: ND-2000C). Spectra of RoxP and the porphyrins alone were likewise recorded.

### Multi-Angle Light Scattering

RoxP was investigated by size-exclusion chromatography in-line with multi-angle light scattering (SEC-MALS) using a Wyatt SEC Analytical Column (Wyatt Technology Europe GmbH, D-56307 Dernbach, Germany, catalog-number: WTC-030N5) connected in-line with an Optilab T-rEX Refractive Index Detector and DAWN 8^+^ Multi-Angle Light Scattering Detector (Wyatt Technology Europe GmbH, D-56307 Dernbach, Germany)

### Confocal Microscopy

Human N/TERT-1 keratinocytes were propagated to confluency on cover slides using keratinocyte serum-free medium (ThermoFisher Scientific, MA 02451, USA, Catalog-number: 17005042) supplemented with 25 μg/mL bovine pituitary extract, 0.2 ng/mL epidermal growth factor, 0.3 mM CaCl_2_ and 100 U/mL penicillin and 100 μg/mL streptomycin. The cells were thoroughly washed with identical medium but without antibiotics before *C. acnes* wildtype or a RoxP deletion mutant was added at a multiplicity of infection of 10. After overnight incubation at 37°C, the medium was removed and the cells were washed three times with PBS supplemented with 1.1 mM Ca^2+^, and 0.5 mM Mg^2+^ before being fixed using 4% paraformaldehyde (ThermoFisher Scientific, MA 02451, USA, Catalog-number: 28906) for 15 minutes. Antibodies from the serum of a rabbit immunized with *C. acnes* lysate (Apronex, 252 42 Jesenice u Prahy, Czech Republic) was purified on a protein A 5 mL HiTrap column (Cytiva, MA 01752, USA, catalog-number: 17040301) according to the manufactures protocol. The resulting 0.9 mg/mL solution of antibodies was diluted 1:500 in PBS and added to the cells. After one-hour incubation, the cells were washed and incubated with a goat anti-rabbit Alexa Fluor 488 antibody (Abcam, Cambridge, UK, Catalog-number: ab150081). After another hour of incubation, the cells were washed initially in PBS then water before being incubated with 1 μM Hoechst 33342 (Abcam, Cambridge, UK, Catalog-number: ab228551) for 15 minutes and subsequently visualized by confocal microscopy using a Zeiss LSM800 Airyscan laser scanning microscope with ZEN 2.3 software (Carl Zeiss, Oberkochen 73447, Germany).

### ABTS Radical Scavenging Assay

3,5 mM 2,2′-azino-bis(3-ethylbenzo-thiazoline-6-sulfonic acid) diammonium salt (ABTS) (Merck, Darmstad, Germany, Catalog-number: A1888) was incubated overnight in the dark with 2.45 mM potassium persulfate (Merck, Darmstad, Germany, Catalog-number: 216244). The solution was diluted with PBS to yield an absorbance of 0.7 at 734 nm and added RoxP in varying concentrations or with peptides from EMC Microcollections (72070 Tübingen, Germany) in a 1:10 molar ratio. Reduction of the ABTS^+*^ radical was monitored by measuring the absorbance at 734 nm and 550 nm (EnSpire) (PerkinElmer, MA01748, USA). For ABTS reduction with peptides, stable absorbance readings were measured after 30 minutes and used as end-point.

### Mass-Spectrometry

RoxP preincubated with 80% (mol/mol) ABTS^+*^ radical for two hours was desalted on two 5 mL desalting columns (Cytiva, MA 01752, USA, catalog-number: 29048684). For intact mass analysis, the protein was initially diluted five-fold in 0.1% trifluoroacetic acid. Equal volumes of protein and 0.1 M 2,5-dihydroxyacetophenone (prepared in 20 mM ammonium dihydrogen citrate containing 75% (v/v) ethanol) were mixed to allow for the formation of a precipitate (Wenzel et al., 2006). 1 μL was subsequently spotted onto a stainless-steel target plate and allowed to dry. The spectra were recorded in positive and linear mode using an AutoFlex Smartbeam III instrument (Bruker Daltronics, Bremen 28359, Germany) calibrated by external calibration (Protein calibration standard I) (Bruker Daltronics, Bremen 28359, Germany). The centroid masses determined were evaluated using the GPMAW software (gpmaw.com). For analysis of the RoxP-ABTS complex, protein was digested over night at 37°C in the presence of 50 mM ammonium bicarbonate by using porcine trypsin in an approximate ratio of 1:20. The digest was reduced by the addition of 50 mM 1,4-dithiothreitol (DTT) for 30 min at room temperature, and subsequently acidified by the addition of 0.1% trifluoroacetic acid (TFA). The digest was applied to reverse-phase separation using by using a BEH300 UPLC C18 column (Waters, 4879 AN Etten-Leur The Netherlands) connected to a Waters Acquity UPLC system. Chromatography was performed by using a linear gradient of solvent B (90% acetonitrile, 0.08% TFA) in solvent A (0.1% TFA) (1% min^-1^). The separation was performed at 40°C using a flow-rate of 300 µl min^-1^. Fractions collected were mixed with α-cyano-4-hydroxycinnamic acid in 60% acetonitrile/0.1% TFA and subjected to MALDI-TOF analysis. The detected ions were subsequently analyzed by MS/MS to identify the peptide collected. The analysis was performed by using an AutoFlex Smartbeam III instrument calibrated by external calibration (Peptide calibration standard I).

## Results

### Porphyrin Binding by RoxP

**Figure 1:**
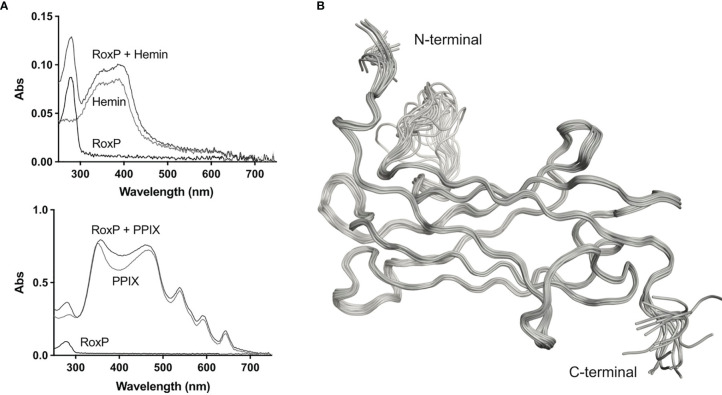
UV-VIS spectroscopy and solution structure of *C. acnes* RoxP. **(A)** UV-VIS scan of recombinant RoxP incubated with either hemin (left) or protoporphyrin IX (PPIX) (right). **(B)** Backbone ribbon traces of the 20 lowest-energy NMR structures calculated.

RoxP has been reported to bind hemin. As this interaction could be pivotal for the antioxidant activity of RoxP, recombinant expression of RoxP in both *E.coli* and *P. pastoris* was initially performed with addition of hemin. However, no signs of hemin binding was observed in the recombinant RoxP. This was also the case for native RoxP purified from *C. acnes* cultures. Hence, we decided to investigate the relevance of porphyrin binding to RoxP by spectral shift in UV/vis absorbance. No spectral shift was observed when hemin was co-incubated with RoxP, indicating a lack of binding. Also the more abundant skin porphyrin, protoporphyrin IX, did not appear to bind RoxP as gauged from this assay (**Figure 1A**). Consequently, subsequent protein expression was performed without the addition of porphyrins.

### Structure Determination of RoxP by NMR

Protein intended for structure determination was produced by bacterial expression of RoxP with its inherent signal peptide (residues 1-23) replaced by a pelB leader sequence which directs RoxP to the bacterial periplasm (Choi and Lee, 2004). Cells were cultured in minimal medium to allow for incorporation of ^13^C and ^15^N, and labeling was found to be 97% efficient as determined by mass spectrometry (data not shown). The structure of RoxP was calculated using 3159 distance constraints of which 1397 were long range. Distance and torsion angle restraints have low average root mean square values of 0.017 Å and 5.2°, respectively, and the structure ensemble has a high precision with a root-mean square deviation (RMSD) for the structured residues (residues 26-136 and 146-160) of 0.22 Å for backbone atoms and 0.49 Å for heavy atoms (**Figure 1B**). The structure is well-determined, with a comprehensive set of Nuclear Overhauser effect (NOE) distance constraints distributed well across the full structure, with the exception of the terminal residues (residues 24-26 and residues 161-164) and one of the loops (residues 137-145). All restraint and structure calculation statistics are shown in **Table 1**.

**Table 1:** Restraint and structure statistics for RoxP.

Restraint statistics	
Distance Restraints^a^	
Total	3159
Intra-residue	579
Sequential	785
Medium range	398
Long range	1397
Dihedral angle Restraint violations: number of/rms	182
Distance violations > 0.1 Å	32/0.017
Torsion violations > 5°	7/5.21
**Structure statistics**	
Coordinate r.m.s.d. to mean: backbone/heavy (Å)^b^	
structured	0.22/0.49
all	0.84/1.27
Angle rms deviation from ideal (°)	0.2
Bond rms deviation from ideal (Å)	0.001
Ramachandran statistics^c^	
Most favoured regions	90.0%
Allowed regions	8.5%
Disallowed regions	1.6%
Structure Quality Validation Metrics^d^ (raw/Z-score)	
Procheck G-factor (phi/psi only)	-0.85/-3.03
Procheck G-factor (all dihedral angles)	-0.83/-4.91
Verify3D	0.19/-4.33
ProsaII (-ve)	0.42/-0.95
MolProbity clashscore	9.92/-0.18

a Long-range meaning that the residue difference, D, was 5 or more and medium range; 1<D<5 (see also **Supplementary Figure 1B**).

b Coordinate rmsd calculated for 20 ensemble members for structured residues: 4-114 and 124-138 and all.

c MolProbity Ramachandran statistics

d Calculated using the Protein Structure Validation Suite (http://psvs-1_5-dev.nesg.org). A positive Z-score indicates a “better” score.

### Solution Structure of RoxP

**Figure 2:**
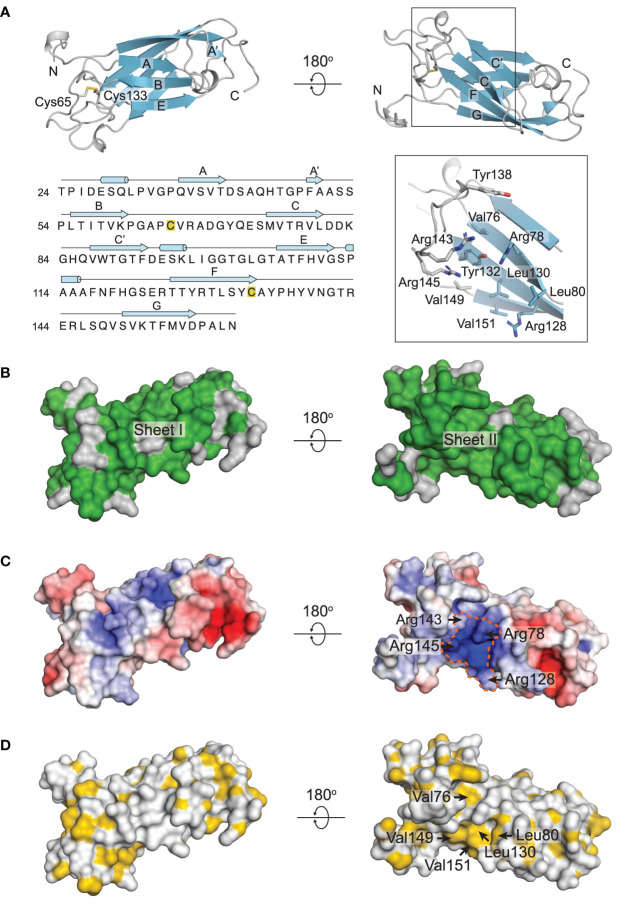
Structural properties of RoxP. **(A)** Cartoon representation of the RoxP two-layered beta-sandwich (colored cyan) comprised of strands A, A’, B and E (sheet I) and C, C’, F, and G (sheet II). The disulfide connecting loop B-C and strand F is shown as sticks. The insert in the lower right panel highlights the groove formed by loop F-G and sheet II. Selected amino acids are shown as sticks. The sequence of RoxP (phylotype I) is shown in the bottom left panel. Secondary structure elements identified in the solution structure of RoxP are shown above the sequence. The two disulfide-forming cysteines are marked with yellow boxes. **(B)** Sequence conservation between RoxP from phylotype I *C. acnes* strains and phylotype II *C. acnes* strains mapped on the surface of RoxP. Identical residues are colored green, while non-identical residues are colored grey. **(C)** Electrostatic potential map mapped on the surface of RoxP with blue representing positive charges and red representing negative charges. The positively charged groove is encircled. **(D)** Hydrophobicity is mapped to a surface representation of RoxP. Hydrophobic groups are colored yellow, while hydrophilic groups are colored white.

The structure of RoxP is composed of a two-layered beta-sandwich (**Figures 1B**, **2A**). The first sheet is comprised of strands A, A’, B and E (sheet I) while the other sheet comprises strands C, C’, F and G (sheet II). Two short alpha-helices can be found in the loops connecting strands C’ and E, and E and F. The N-terminal stretch preceding strand A likewise features a short helix. The overall fold is further stabilized by a disulfide bond between Cys65 of loop B-C and Cys133 of strand F (**Figure 2A**). RoxP resembles the constant domain of an antibody with a beta-strand arrangement A-A’-B-C-C’-E-F-G, when following the polypeptide backbone, and is best classified as an immunoglobulin-like (Ig-like) I2-set topology (**Supplementary Figure 1**). The RoxP beta-strands are connected with relatively long and rigid loop regions, both within the same sheet and across sheets, with several loops containing multiple beta-turns and hydrogen bonds. The F-G loop is, however, more flexible and contains several backbone resonances for residues 137-145 that could not be assigned or display much weaker intensities, possibly indicating dynamics on the microsecond time scale. This segment features no long-range nor medium-range NOEs and, consequently, the local structure of the loop is determined with lower precision. This in contrast to loop E-F, which is rigid as suggested by the significant dispersion of the assigned side-chain resonances and by deviation from random-coil chemical shifts. Backbone dihedral angle constraints could be obtained by chemical shift and sequence homology inference by TALOS-N for residues Tyr138 and Thr142, which revealed extended backbone conformations.

Ig-like domains show great variation in their amino acid sequences, but can be aligned based on their three-dimensional structures. This reveals trends in the residues constituting the hydrophobic core of the domains (Halaby et al., 1999). Although RoxP belongs to the I2-set domains, it follows the hydrophobicity pattern found in C2-set domains, except for Thr77 and Thr126 (**Supplementary Figure 2**). In phylotype II strains, however, Thr77 is alanine, thereby adhering to the hydrophobic pattern at this position. The topological difference between C2-set and I2-set domains is a short A’ strand that forms hydrogen bonds to strand G and thereby extends sheet II (**Supplementary Figure 1**). This is also true for RoxP where the A’ strand appears to be rather short and only two backbone hydrogen bonds are formed (Phe49-Asp157 and Ala51-Asp175) between strands A’ and G. RoxP from phylotype I *C. acnes* strains and phylotype II *C. acnes* strains have 83% sequence identity (**Supplementary Figure 3**). Their three-dimensional structures are therefore expected to be almost identical. Most of the amino acid differences between RoxP from phylotype I *C. acnes* strains and phylotype II *C. acnes* strains are positioned at the face constituted by sheet I, whereas the opposite face constituting sheet II is almost fully conserved (**Figure 2B**). Notably, the region containing the flexible loop that connects strands F and G and forms a small groove together with sheet II is conserved, except for a conservative threonine to serine substitution. An electrostatic-potential map shows this groove to display pronounced positive charge (**Figure 2C**). The positive potential results from the closely spaced arginine residues, Arg78 and Arg128 of sheet II, and Arg143 and Arg145 positioned in the F-G loop (**Figure 2A, C**). Besides the arginine residues, the groove also contains several outward-facing hydrophobic residues (Val76, Leu80, Leu130, Val149, and Val151) in addition to two tyrosine residues, Tyr132 and Tyr138, of which the former is placed deep in the groove while the latter is found at the top of the perpendicular F-G loop (**Figures 2A, D**). Overall, this suggests that the groove could be functionally important and be a site for interaction with a yet unidentified anionic and/or hydrophobic binding partner.

**Figure 3:**
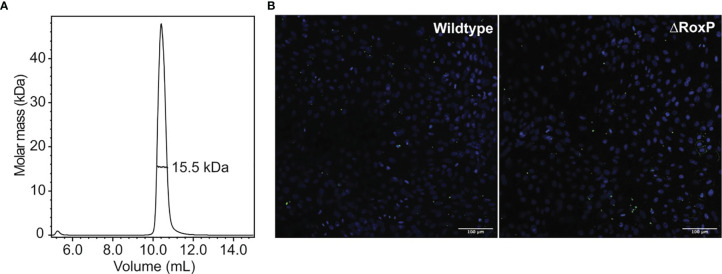
RoxP oligomerization and role in cell adhesion. **(A)** Oligomerization analysis of recombinant RoxP by size-exclusion chromatography with inline multi-angle light scattering. **(B)** Representative confocal microscopy images of adhesion of *C. acnes* wildtype (left) and *C. acnes* RoxP deletion strain (right) to N/TERT-1 keratinocytes (nucleus colored blue, bacteria colored green). Scale bar is 100 μm.

To identify proteins with structural homology, the structure of RoxP was submitted to the DALI server (Holm, 2020) and compared to a representative set of deposited protein structures (PDB25). This revealed no distinct hits, but rather a comprehensive list of functionally diverse proteins containing Ig-like domains, emphasizing that this domain is common and found throughout protein classes with diverse functions. The majority of structural homologues appeared to be involved in cell adhesion (**Supplementary Figure 4**). RoxP is a secreted protein, but cell-wall association of RoxP has been reported (Yu et al., 2015). Involvement in cell adhesion would therefore match its physiological location. Adhesion proteins are often formed by consecutive Ig-like domains that form long and protracted structures (Jones et al., 1992; Harrison et al., 2012; Ozkan et al., 2014; Pronker et al., 2016). We therefore speculated if RoxP could be involved in host-cell adhesion, possibly by assembling into an oligomeric, elongated structure. However, no sign of oligomerization was evident in the structure calculation or from size-exclusion chromatography with inline multi-angle light scattering (**Figure 3A**). In principle, multimerization of RoxP could depend on the presence of a binding partner or be hindered by the experimental conditions that differ from the lipid-rich milieu in sebaceous follicles. To test involvement of RoxP in host cell interaction, *C. acnes* and a *C. acnes* RoxP deletion strain (Allhorn et al., 2016) were co-cultured with N/TERT-1 keratinocytes (Dickson et al., 2000) and subsequently visualized by confocal microscopy (**Figure 3B**). Here, both adherence of wildtype and RoxP deletion mutant to keratinocytes was observed after extensive washing, suggesting that RoxP does not play a role in adherence to these cells. This, however, does not eliminate the possibility of involvement in adhesion to other cells, such as sebocytes or that an unknown ligand or factor is absent from the experimental conditions.

### Reduction of ABTS-Radicals Is Mediated by Tyrosine Residues

**Figure 4:**
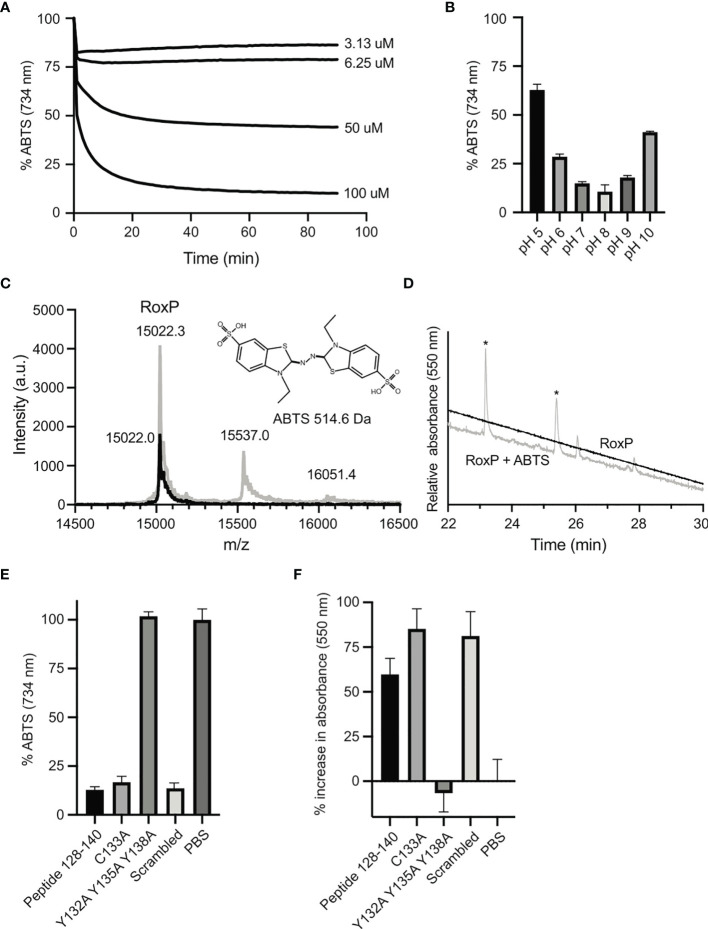
Antioxidant activity of RoxP. **(A)** The radical-scavenging ability of RoxP was assayed by measuring the amount of ABTS^+*^ using different concentrations of RoxP. **(B)** The pH-dependency of ABTS^+*^ reduction was monitored at pH 5 to 10. **(C)** Intact mass spectrometry of RoxP (black line) or RoxP incubated with 80% (mol/mol) ABTS^+*^ radical (grey line). **(D)** Separation of trypsin-treated RoxP-ABTS complex (grey line) or RoxP (black line) on a reverse-phase column monitored at 550 nm. **(E)** Relative reduction of ABTS^+*^ radicals by residues 128-140 and variants thereof followed spectrophotometrically at 724 nm. The absorbance measured with PBS is set to 100%. **(F)** Reduction of ABTS^+*^ radicals by residues 128-140 and variants thereof followed spectrophotometrically at 550 nm. Increase in absorbance is normalized to measurements with PBS.

RoxP has been attributed antioxidant activity as ABTS^+*^ radicals are readily reduced by RoxP (Allhorn et al., 2016). To further investigate this, reduction of ABTS^+*^ was followed over time using different RoxP concentrations (**Figure 4A**). The reduction was saturable, suggesting that a regenerative mechanism is absent. The reduction was most efficient close to neutral pH, but even at pH 5 and 10 a large proportion of ABTS^+*^ was reduced (**Figure 4B**). ABTS^+*^ reduction is routinely measured as decreasing absorbance at 734 nm as the radical is converted into a colorless product. Interestingly, upon reduction, a product with a strong purple color absorbing at 550 nm was generated. It is well-documented that both the ABTS di-cation and adducts formed between ABTS^+*^ radicals and phenolic compounds absorb at 550 nm (Kahkonen et al., 1999; Ilyasov et al., 2020). The purple product suggested either a covalent bond between ABTS and RoxP or a strong non-covalent interaction to the ABTS di-cation. To explore this further, the RoxP-ABTS complex was investigated by intact mass spectrometry (**Figure 4C**). Both the RoxP-ABTS sample and RoxP alone, showed a peak with a mass of 15022.3 Da. Two other peaks were seen at 15537.0 Da and 16051.4 Da, and perfectly match one or two ABTS moieties coordinated to RoxP, respectively. To examine the nature of this interaction, the RoxP-ABTS complex was digested with trypsin and separated on a reverse phase column (**Figure 4D**). Two peaks with absorbance at 550 nm were seen and identified by mass spectrometry as the peptides GYQESMVTR (residues 70-78) and the non-tryptic peptide TLSYCAYPH (residues 129-137). Residues 129-137 form strand F and part of the F-G loop while residues 70-78 form strand C and part of the B-C loop, both regions are thus positioned near the groove formed between loop F-G and sheet II. Formation of a purple compound was also reported for the protein lipocalin alpha1-microglobulin incubated with ABTS^+*^ (Akerstrom et al., 2007). Here, a covalent bond is formed between an ABTS fragment and a tyrosine residue in a cysteine-dependent mechanism. To further investigate the reaction between RoxP and the ABTS^+*^ radical, and to see if a similar mechanism is at play, a peptide corresponding to residues 128-140 was synthesized. Furthermore, to determine the effect of the cysteine residue, the tyrosine residues and any positional dependencies, the three peptide variants C133A, Y132A Y135A Y138A, and a scrambled peptide variant were likewise synthesized. The C133A variant along with the scrambled peptide showed similar reductive capabilities as the native peptide (**Figure 4E**), while the peptide with all three tyrosine residues mutated to alanine showed no reduction. In agreement with this, formation of the purple product showed inverse correlation with the ABTS^+*^ radical concentration (**Figure 4F**). In conclusion, the reduction of ABTS^+*^ appears to be dependent on the tyrosine residues irrespective of their position in the peptide, indicating a non-specific, and non-enzymatic reduction.

## Discussion

A lot of attention has been devoted to the potential role of *C. acnes* in the common skin disorder acne vulgaris (Dreno et al., 2018; O'Neill and Gallo, 2018; McLaughlin et al., 2019). Despite a long history of research, to date, it cannot be concluded if *C. acnes* is an important driver or a passive bystander in acne. To understand the role of *C. acnes* in both disease and health, it is essential to understand both the deleterious and beneficial effects caused by this bacterium. Earlier studies have found that RoxP is the most-secreted protein of cultured *C. acnes*, suggesting that it has an important function (Holland et al., 2010). Moreover, and importantly, RoxP was found to be produced *in vivo* on healthy skin (Bek-Thomsen et al., 2014; Erturk Bergdahl et al., 2019). The protein was reported to be essential for bacterial growth under oxic conditions as well as essential for colonization of skin in an *ex vivo* model (Allhorn et al., 2016; Andersson et al., 2019). The underlying mechanisms for these observations and thus the function of RoxP is, however, difficult to deduce from the knowledge obtained hitherto. To shed light on the function of RoxP, we established a highly efficient bacterial expression protocol. A periplasmic localisation signal was found essential for expression as all attempts at cytoplasmic expression failed. Presumably, formation of the disulfide bond between loop B-C and strand F is pivotal for correct folding. Structure calculation by NMR spectroscopy revealed a core Ig-like domain. At first glance, the domain could be classified as a C2-set. However, a short A’ strand, spanning residues Phe49 and Ala50, was found in all 20 calculated NMR-structures by DSSP (Kabsch and Sander, 1983). Consequently, the domain is classified as an I2-set domain. The Ig-like domain belongs to the so-called “superfold” configurations; an energetically favorable fold which explains its widespread occurrence in proteins with diverse functionalities (Orengo et al., 1994; Steiner, 1996) and a consensus sequence for these proteins is not a prerequisite for adopting a similar overall topology and shape (Halaby et al., 1999). Many proteins involved in cell adhesion, such as integrins, utilize Ig-like domains to form elongated structures that can bind host cell components (Wang et al., 1995; Recacha et al., 2014). However, we did not find evidence for multimerization of RoxP, and were neither able to detect any differences in bacterial adhesion to keratinocytes between wildtype *C. acnes* and a *C. acnes* RoxP deletion strain. Here it should be noted, however, that the experimental conditions used were not identical to the sebum-rich environment of RoxP, which could affect its behavior. In this regards, the high water-solubility of RoxP is surprising when considering its natural lipophilic environment (Picardo et al., 2009). RoxP was originally reported to bind heme and the groove formed by sheet II and loop F-G could possibly accommodate a porphyrin moiety. A mechanism to capture free porphyrins would be physiologically relevant as *C. acnes* is a porphyrin producer, and excess of porphyrin, in combination with UV-light exposure, can lead to skin barrier damage (Johnson et al., 2016; Barnard et al., 2020); thus, a bacterial mechanism to prevent porphyrin-mediated skin inflammation would be highly beneficial. We did, however, not detect interaction between RoxP and heme, or to the more skin-abundant protoporphyrin IX (Lee et al., 1978; Johnson et al., 2016) by UV-VIS. Heme is notoriously sticky and often leads to false-positive binding. This may potentially have been a factor in earlier reports of heme-binding (Allhorn et al., 2016). Irrespectively, the positively charged groove of sheet II and loop F-G may accommodate a similar-sized, negatively charged cofactor or substrate.

The recombinantly produced RoxP showed identical antioxidant activity as to naturally produced RoxP and also to RoxP produced in yeast (Andersson et al., 2019). Detection and quantification of redox activity is routinely done using the synthetic ABTS^+*^ radical (Miller and Rice-Evans, 1997). ABTS does, however, not resemble any physiologically relevant molecule and so conclusions on antioxidant activity using this assay should be made with caution (Tirzitis and Bartosz, 2010). This is especially true for RoxP as the saline buffer conditions used in the ABTS assay differ markedly from the lipid-rich environment in the follicles. By mass spectrometry, we identified two RoxP fragments that interact with ABTS. Considering the harsh conditions utilized for MS/MS, which would abrogate many non-covalent interactions, it is surprising that no covalent bonds between ABTS and RoxP were detected. We did, however, only detect weak absorbance at 550 nm for the tryptic RoxP-ABTS complexes during purification. Synthetic peptide variants corresponding to residues 128-140 revealed that the antioxidant activity is likely independent of Cys133 and could solely resides with the tyrosine residues, irrespective of their position in the peptide. Lack of cysteine-involvement was expected as the cysteine residue, unlike in the lipocalin alpha1-microglobulin, is engaged in a disulfide bond with Cys65 and so is not available to participate in the reaction. The reaction could, however, be promoted by the closely located arginine residues, which may function to stabilize a negatively charged phenolate ion and thereby enhance the reactivity between tyrosine and radicals (Litwinienko and Ingold, 2007). Reduction of ABTS with peptides reached a plateau after 15 minutes. This is in good agreement with Allhorn et al. (2016), who reported a plateau with native RoxP after 12 minutes, indicating that the same mechanism is at play. The two peptides detected by absorbance at 550 and identified by MS/MS only comprise three out of five tyrosines in RoxP. From the structure it can be seen that Tyr127 is likely sterically blocked from interacting with the ABTS radical. Tyr138 should be readily available as it is positioned in a loop. However, interaction between nearby tyrosines and ABTS could induce conformational changes to the loop thereby positioning Tyr138 less favorably for interacting with ABTS possibly pushing the degree of modification below the detection limit. Intriguingly, both RoxP and the peptides elicit reductive capabilities beyond a one to one molar ratio. This could indicate that regeneration takes place and that the interaction between RoxP and ABTS is transient, which fits with the mass spectrometric experiments where no covalent bond between RoxP and ABTS was found. Although not fully understood, the reaction with ABTS does imply a mechanism involving electron transfer. Such a transfer might involve a yet-to-be-found cofactor fitting into the speculative binding pocket. Considering that the *C. acnes* RoxP deletion strain grows significantly slower under oxic conditions than wildtype bacteria, it can be postulated that RoxP is involved in respiration by partaking in electron transfer to molecular oxygen. In this regard, it is intriguing that genes encoding enzymes involved in the biosynthesis of menaquinone, a molecule involved in electron transport, can be found immediately upstream and downstream of the *roxP* gene. Further studies looking at alterations in the biosynthesis of menaquinone e.g. by transcriptomic analysis of wildtype *C. acnes* and the *C. acnes* RoxP deletion strain could be highly interesting. Although RoxP plays a role in bacterial growth when oxygen is present, it is difficult to elucidate if this owes to a function in metabolism, neutralizing harmful substances, respiration or something else. Thus, to get closer to understanding the function of RoxP, it is imperative to find specific processes that are affected by the presence of RoxP. If RoxP does in fact serve as an antioxidant on the skin, this opens the possibility of using RoxP in novel treatment strategies aimed to alleviate oxidative stress in skin diseases. The feasibility of such an approach is substantiated by the observation of a decline in RoxP concentration in skin affected by oxidative diseases (Andersson et al., 2019). The method for high yield expression of RoxP described here, could be important for utilization of RoxP in these treatment strategies.

In conclusion, we show that RoxP adopts a compact, Ig-like domain, which explains its pronounced stability (Andersson et al., 2019). We furthermore show that the antioxidant activity is harbored by tyrosine residues, and likely constitutes only part of the function of RoxP - a function which may depend on a highly positively charged groove, containing several solvent-exposed, hydrophobic residues. The structure determined here will be of great value as a framework for future studies on elucidation the function of RoxP while also adding to the general knowledge of the Ig-like domain family.

## Data Availability Statement

The atomic coordinates of RoxP has been deposited in the protein databank (http://wwpdb.org/ PDB:7BCJ). NMR assignments have been deposited in the BioMagResBank (https://bmrb.io) under accession number 34585.

## Author Contributions

KS, BP, HB, and CA designed and performed the experiments. SP performed the mass spectrometry analysis. JN and FM performed the NMR experiments and structure calculations. KS, JN, HB, and CA wrote the manuscript. All authors read and approved the final manuscript.

## Funding

This work was supported by the Novo Nordisk foundation (grant reference numbers NNF18OC0053172 and NNF16OC0022788) and the Interreg Europe, MAX4ESSFUN project (grant reference number AU-005). The Danish Center for Ultrahigh-Field NMR spectroscopy is supported by the Ministry of Higher Education and Science (grant reference number AU-2010-612-181).

## Conflict of Interest

BP is employed by S-biomedic.

The remaining authors declare that the research was conducted in the absence of any commercial or financial relationships that could be construed as a potential conflict of interest.

## Publisher’s Note

All claims expressed in this article are solely those of the authors and do not necessarily represent those of their affiliated organizations, or those of the publisher, the editors and the reviewers. Any product that may be evaluated in this article, or claim that may be made by its manufacturer, is not guaranteed or endorsed by the publisher.

## Abbreviations

ABTS, 2,2′-azino-bis(3-ethylbenzothiazoline-6-sulfonic acid) diammonium salt; *C. acnes*, *Cutibacterium acnes*; DTT, 1,4-dithiothreitol; EDTA, Ethylenediaminetetraacetic acid; Ig-like, Immunoglobulin-like; IPTG, isopropyl beta-D-1-thiogalactopyranoside; PBS, Phosphate-buffered saline; RMSD, Root-mean-square deviation; ROS, Reactive oxygen species; RoxP, Radical oxygenase of *Propionibacterium acnes*; SEC-MALS, Size-exclusion chromatography multiple angle light scattering; TFA, Trifluoroacetic acid; UV-VIS, Ultraviolet-Visible.
