# Supplementary material for: Solution Structure of the Cutibacterium acnes-Specific Protein RoxP and Insights Into Its Antioxidant Activity
Source: Front Cell Infect Microbiol. 2022 Feb 11;12:803004. doi: 10.3389/fcimb.2022.803004 (PMC8873378; doi:10.3389/fcimb.2022.803004)
Supplement: Supplementary file 1 [file DataSheet_1.docx]

**Supplementary Figure 1:**


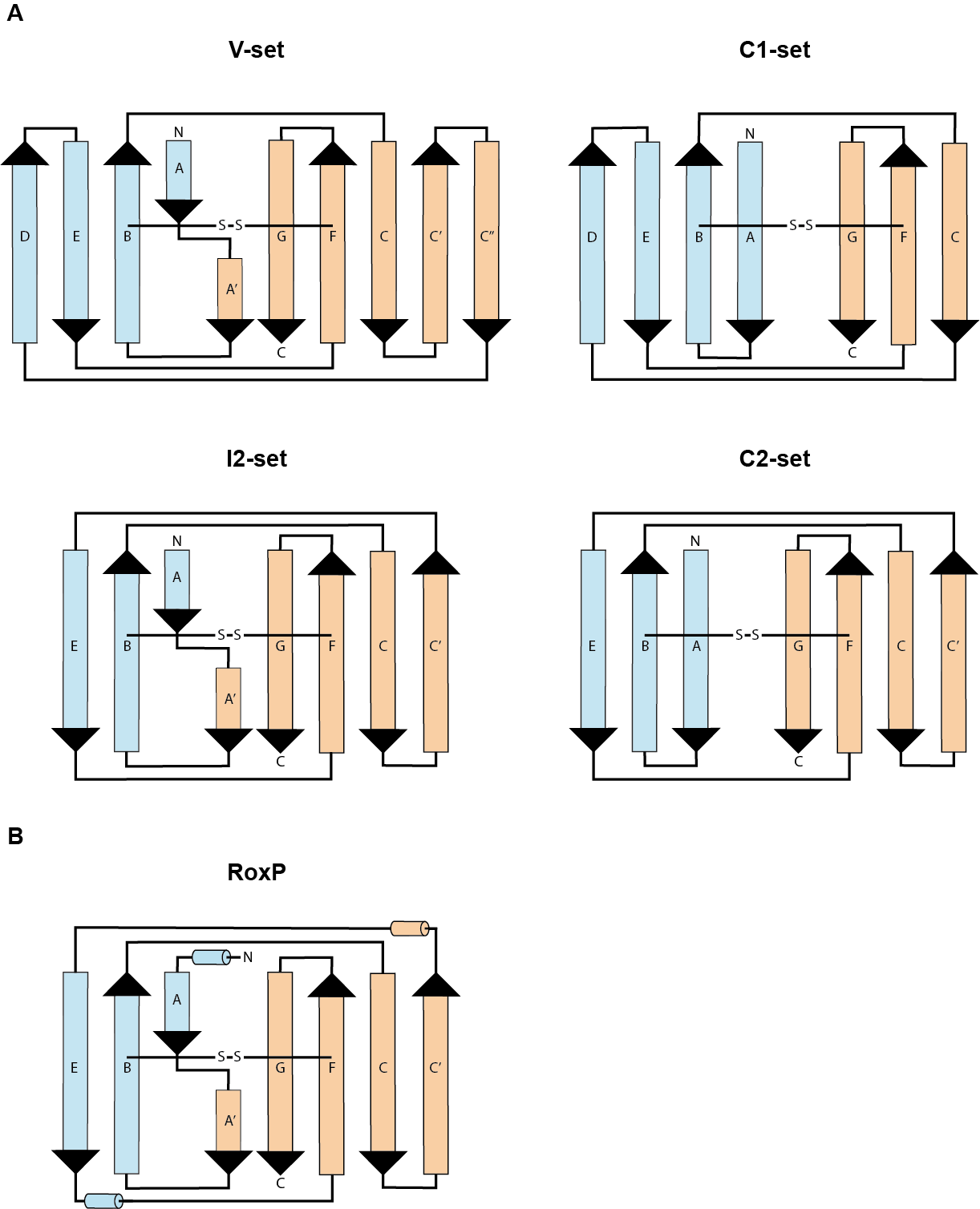


**Supplementary Figure 1: Ig-like folds and RoxP. A)** Topology diagrams of common sets of Ig-like folds. **B)** Topology diagram of RoxP. Sheet I is colored blue, while sheet II is colored beige. Beta-strands are shown as arrows and alpha-helices are shown as cylinders.

**Supplementary Figure 2:**


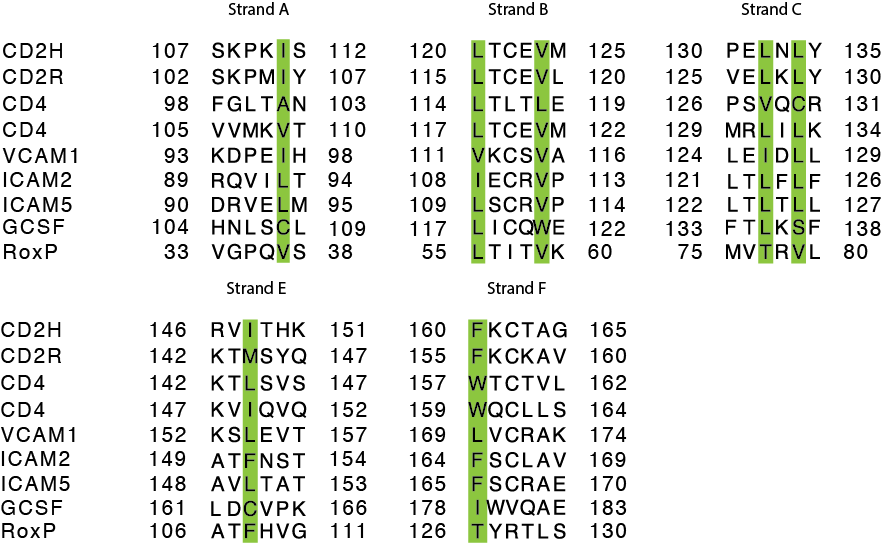


**Supplementary Figure 2: Structure-based sequence alignment of C2-set domains and RoxP.**

Conserved topohydrophobic positions are marked in green. (CD2H: Cluster of Differentiation 2 Human, CD2R: Cluster of Differentiation 2 Rat, VCAM: Vascular Cell Adhesion Molecule, ICAM: Intercellular Adhesion Molecule, GCSF: Granulocyte Colony-stimulating Factor, D: Domain)

**Supplementary Figure 3:**


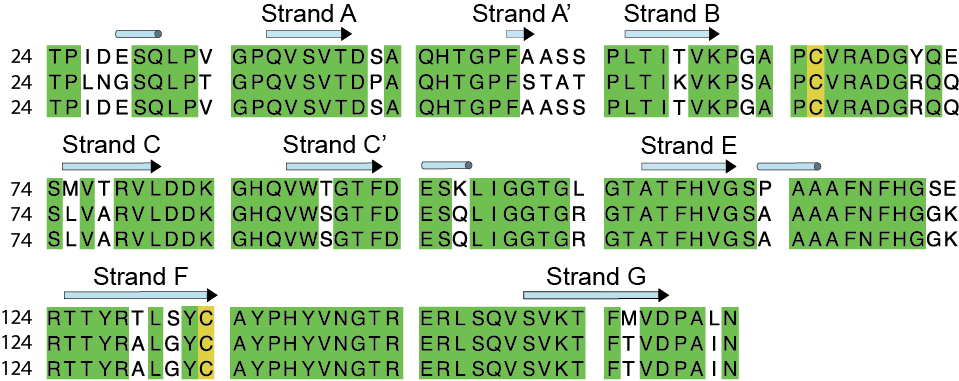


**Supplementary Figure 3: Sequence alignment of RoxP variants.** Sequence alignment of three RoxP variants (Uniprot accessions I6PPR9, I6PNA2, I6PHU9). Conserved residues are marked with green boxes. Cysteines are marked with yellow boxes. Secondary structure elements identified in the solution structure of RoxP are shown above the sequences.

**Supplementary Figure 4:**

**Supplementary Figure 4: Functional distribution of RoxP structural homologues identified by the Dali server.** The Dali server identified 284 structural homologous of RoxP with a Z-score ≥ 5.0, which were classified according to biological function.
